# *TomocuPy* – efficient GPU-based tomographic reconstruction with asynchronous data processing

**DOI:** 10.1107/S1600577522010311

**Published:** 2023-01-01

**Authors:** Viktor Nikitin

**Affiliations:** a Advanced Photon Source, Argonne National Laboratory, Lemont, IL 60439, USA; Tohoku University, Japan

**Keywords:** tomography, reconstruction, GPU, NVMe SSD, conveyor processing, asynchronous processing

## Abstract

Fast 3D data analysis and steering of a tomographic experiment by changing environmental conditions or acquisition parameters require fast, close to real-time, 3D reconstruction of large data volumes. Here a performance-optimized *TomocuPy* package is presented as a GPU alternative to the commonly used central processing unit (CPU) based *TomoPy* package for tomographic reconstruction. *TomocuPy* utilizes modern hardware capabilities to organize a 3D asynchronous reconstruction involving parallel read/write operations with storage drives, CPU–GPU data transfers, and GPU computations. In the asynchronous reconstruction, all the operations are timely overlapped to almost fully hide all data management time. Since most cameras work with less than 16-bit digital output, the memory usage and processing speed are furthermore optimized by using 16-bit floating-point arithmetic. As a result, 3D reconstruction with *TomocuPy* became 20–30 times faster than its multi-threaded CPU equivalent. Full reconstruction (including read/write operations and methods initialization) of a 2048^3^ tomographic volume takes less than 7 s on a single Nvidia Tesla A100 and PCIe 4.0 NVMe SSD, and scales almost linearly increasing the data size. To simplify operation at synchrotron beamlines, *TomocuPy* provides an easy-to-use command-line interface. Efficacy of the package was demonstrated during a tomographic experiment on gas-hydrate formation in porous samples, where a steering option was implemented as a lens-changing mechanism for zooming to regions of interest.

## Abstract 2

*TomocuPy*, a Python software package for fast 3D reconstruction on graphics processing units (GPUs) and modern storage drives supporting parallel read/write operations, is presented. The package demonstrates significant performance gain compared with analogs and can be efficiently used when processing data during experiments requiring steering of environment conditions and acquisition schemes, or triggering data capturing processes for other measuring devices.

## 1. Introduction

Fast *in situ* tomographic experiments at synchrotron facilities are of great interest to various user communities including geology (Butler *et al.*, 2020 ▸; Nikitin *et al.*, 2020 ▸), material science (Maire *et al.*, 2016 ▸; Zhai *et al.*, 2019 ▸) and energy research (Finegan *et al.*, 2015 ▸; Liu *et al.*, 2019 ▸). This is because modern synchrotron light sources of the third and fourth generation provide the necessary photon flux to accommodate very fast scanning of large samples with micrometre and nanometre spatial resolution (Willmott, 2019 ▸; De Andrade *et al.*, 2021 ▸; Nikitin *et al.*, 2022 ▸). At the same time, modern detectors allow for continuous tomographic data acquisition at more than 7.7 GB s^−1^ rate (Mokso *et al.*, 2017 ▸; García-Moreno *et al.*, 2021 ▸), generating a series of tomographic datasets representing dynamic sample states at unprecedented high temporal resolution.

One of the most challenging tasks nowadays is the efficient steering of such dynamic experiments. Both manual and AI-based experiment steering can be performed more efficiently utilizing full 3D reconstructed volumes rather than projection images. Reconstructed volumes are more informative and contain all information about the current sample state compared with the projection raw data or a subset of reconstructed slices. Therefore, the performance of 3D tomographic reconstruction is critical when processing large amounts of data captured in a short period of time. Fast, close to real-time 3D reconstruction will allow for AI data analysis and steering experiments, *e.g.* by automatic changing of environmental conditions (pressure, temperature, *etc*.) or by triggering data capturing processes for other measuring devices (higher resolution or ultrafast detectors).

Besides fast reconstruction of dynamic tomography data, there is still a need to accelerate the processing of large datasets, in particular those obtained from detectors with large sensors or from mosaic scans. In mosaic scans, large samples are scanned at different vertical and horizontal positions to obtain a set of datasets that are then stitched together to generate one large dataset. 3D reconstructions of such large datasets can have more than 10k voxels in each dimension, yielding several Tb of data to process (Vescovi *et al.*, 2017 ▸; Borisova *et al.*, 2021 ▸). To obtain reconstruction of such datasets in a reasonable time, tomography software packages are typically adapted for high-performance computing (HPC) clusters [see, for instance, Hidayetoğlu *et al.* (2020 ▸) and references therein]. It is common that data analysis by regular beamline users is delayed due to the lack of immediate access to such HPC clusters.

Nowadays there exist many packages for tomography data reconstruction. *TomoPy* (Gürsoy *et al.*, 2014 ▸) provides a Python interface for pre-processing tomography data and for applying filtered backprojection to recover 3D sample volumes with parallel-beam geometry. It provides implementations of different reconstruction methods, including *Gridrec* (Dowd *et al.*, 1999 ▸; Rivers, 2012 ▸) amongst others (Gürsoy, 2014 ▸), additionally accelerated with central processing unit (CPU) multiprocessing, Intel compiler directives, and Intel Math Kernel Library (MKL). The computational complexity for reconstructing a 3D volume is 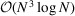 , assuming that the number of projection angles and volume size in each dimension are of the order of *N*. The *Gridrec* implementation on computer clusters is also available (Marone *et al.*, 2017 ▸) and has demonstrated first steps towards on-the-fly tomography data processing. *TomoPy* supports Python wrappers to run reconstruction functions from other packages. One example of such a wrapper is *ASTRA Tomography Toolbox* (van Aarle *et al.*, 2015 ▸, 2016 ▸), which is also commonly used as an independent package. The *ASTRA Toolbox* implements high-performance graphics processing unit (GPU) primitives not only for parallel-beam tomography but also for cone-beam tomography. Besides the regular filtered backprojection method based on the summation over lines [ 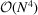 computational complexity], the package is optimized to work with iterative reconstruction methods such as SART (Andersen & Kak, 1984 ▸), SIRT (Gregor & Benson, 2008 ▸) and CGLS (Scales, 1987 ▸). For an iterative method, it is possible to keep all the necessary data in the GPU memory, and thereby reduce the data copy between the storage drive, CPU and GPU memory. In such cases, the performance of reconstruction is mostly limited by GPU computation speed. Another package, called *UFO* (Vogelgesang *et al.*, 2016 ▸), provides a multi-threaded, GPU-enabled and distributed data processing framework. Tomographic and laminographic reconstructions are also implemented using the regular filtered backprojection method of complexity 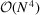 .

Computational complexity plays an important role when reconstructing data from large detectors, or from data obtained by stitching several projection datasets (Vescovi *et al.*, 2018 ▸; Tile, 2022 ▸). For example, for *N* = 2048 the complexity 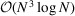 becomes approximately 186 times lower than 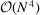 . With increasing data sizes, the potential acceleration becomes higher (341 complexity lowering factor for *N* = 4096, 630 for *N* = 8192, and so on). Therefore it is always beneficial to operate with algorithms of lower computational complexity with the introduction of new detectors having large sensors (*e.g.* 13392 × 9528 sensor shr661 camera from SVS-VISTEK); they indeed become critical for any future tomography applications. Examples of methods with 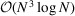 complexity include the Fourier-based gridding method (Beylkin, 1998 ▸) and the log-polar-based method (Andersson *et al.*, 2016 ▸). In contrast, methods implemented in the *ASTRA* and *UFO* packages have 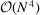 computational complexity and therefore become less efficient when processing data from huge detectors.

In this work, we present a new package called *TomocuPy* where we combined efficient reconstruction methods and modern hardware capabilities to accelerate the whole tomographic reconstruction process, including data read/write operations with storage drives, CPU–GPU data transfers, and computations on GPU. The main features of the packages include:

(1) *Optimized GPU implementation of reconstruction with low [ 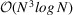 ] computational complexity (Fourier-based gridding method and log-polar-based method)*. The methods were developed previously; however, they have not been commonly used as a regular tool inside a tomographic package such as *TomocuPy*. The performance table of Andersson *et al.* (2016 ▸) reports 0.045 s log-polar-based reconstruction of one slice, 2048 × 2048, on Nvidia GeForce GTX 770 (release date: 30 May 2013), which corresponds to 92 s for reconstructing the full volume. The reported time does not include initialization and data transfer costs. Modern GPUs are several tens of times faster than GTX 770 and reduce reconstruction times to a few seconds.

(2) *Asynchronous chunk data processing where read/write operations with storage drives, CPU-GPU data transfers, and GPU computations for each chunk are timely overlapped.* It is known that one of the main bottlenecks slowing down reconstruction when using GPUs is data management. Computations on GPUs may take less time than data read/write from storage drives and CPU–GPU data transfers. *TomocuPy* provides the functionality to almost fully hide time for all data management. In this work, we optimize operation with modern storage based on Non-Volatile Memory Express (NVMe) solid state disks (SSDs). They deliver unprecedented performance provided by parallelization of the read/write operations, which results in 8× acceleration compared with regular SATA SSDs (Xu *et al.*, 2015 ▸). Besides computer clusters, current NVMe SSDs connected via PCIe v3 or PCIe v4 are also used in common workstations and demonstrate 3.5–7 GB s^−1^ speed for parallel operations with the disk. In this case, writing one tomographic volume of size 2048^3^ in 32-bit precision may potentially take less than 5 s.

(3) *16-bit (half-precision) arithmetic.* Most detectors used for tomography have less than 16-bit digital output. It is therefore potentially possible to decrease processing data sizes and accelerate computations even more. *TomocuPy* implements all processing methods in both 16- and 32-bit precision. 16-bit computations decrease reconstruction sizes, accelerate computations and demonstrate acceptable accuracy for processing experimental datasets.

(4) *Command-line interface for reconstruction.* *TomocuPy* provides a command-line interface for processing tomographic datasets stored in the HDF5 format. The interface includes necessary commands and parameters for tomographic data pre-processing and reconstruction. It is also easy to extend the interface by adding new functionality with a description of parameters.

The rest of the paper is organized as follows. In Section 2 we describe the implementation details and an easy-to-use command-line interface for processing experimental data. Section 3 provides performance analysis on synthetic data and accuracy analysis on experimental data from a micro-CT synchrotron beamline and a comparison between different methods. An example of a dynamic tomography experiment at a synchrotron where automatic steering was possible due to fast reconstruction provided by *TomocuPy* is presented in Section 4. Conclusions and outlook are given in Section 5.

## 2. Fast GPU-based reconstruction with *TomocuPy*

*TomocuPy* (Nikitin, 2022 ▸) is a Python package that provides support for fast and efficient asynchronous data management and tomographic reconstruction on Nvidia GPUs with 16-bit or 32-bit computational precision. It implements GPU-based pre-processing steps and filtered backprojection operators, as well as optimized data transfer mechanisms among storage drives, CPU RAM memory and GPU memory. In the following we will describe the main package features leading to fast, close to real-time, tomographic reconstruction.

### 2.1. 16-bit precision arithmetic

Area detectors used for tomographic imaging incorporate an analog-to-digital converter (ADC) to digitize the images with 8-, 10-, 12- or 16-bit output. The conventional tomography reconstruction is typically performed with 32-bit floating-point operation which might be inefficient in terms of computational speed. In this work we considered 16-bit floating-point (FP16) arithmetic as an alternative to the conventional 32-bit floating-point (FP32) arithmetic. FP16 is used in many computer graphics environments to store pixels, including Nvidia CUDA, OpenGL and Direct3D. Currently, it is also gaining popularity in deep learning applications with Nvidia GPUs. Nvidia’s recent Pascal architecture was the first GPU that offered FP16 support. FP16 arithmetic was significantly optimized for following Nvidia architectures including Volta and Ampere, and became beneficial for code optimization in terms of performance and memory usage.

To adapt tomographic reconstruction in *TomocuPy* for FP16 computations we followed the guidance from Ho & Wong (2017 ▸) that shows different issues and opportunities with code migration to FP16. We also reviewed all mathematical operations in the code and made sure that the accuracy and correctness of computations are not lost. The accuracy can be lost when a mathematical operation is performed between large and small numbers, *e.g.* 1000 − 0.1 = 1000 (FP16). Incorrect results are obtained when multiplying two large numbers: 1000 × 1000 = inf (FP16) since the maximum representable value in FP16 precision is 65504. To address these issues, we reorganized arithmetic operations where possible. In places where reorganization was not possible, we performed the operation with arguments converted to FP32 and cut the precision of the result back to FP16. As a result, we were able to decrease the total amount of memory (CPU RAM, GPU and storage disk space) by two times and accelerate computations on GPU.

### 2.2. Pre-processing steps and backprojection

Pre-processing steps in tomographic reconstruction include dark/flat-field correction, taking a negative logarithm of the data, and one-dimensional filtering with the Shepp–Logan, Parzen or other filter. Additionally, pre-processing may include ring-removal filtering using wavelets (Münch *et al.*, 2009 ▸) or by analytical formula (Titarenko *et al.*, 2010 ▸; Titarenko, 2016 ▸), zinger artifacts reduction (Rivers, 1998 ▸) and the propagation-based phase-retrieval procedure using the Paganin filter (Paganin *et al.*, 2002 ▸). To accelerate computation of all these steps we used the *CuPy* Python library (Okuta *et al.*, 2017 ▸), which is a GPU-accelerated analog of the *NumPy* Python library. All regular linear algebra operations, such as multiplication, summation, logarithm, exponent, are easily ported to *CuPy* library calls in 16-bit and 32-bit floating-point precision. At the time of writing, *CuPy* does not support computing fast Fourier transforms (FFTs) in 16-bit precision; moreover, 16-bit FFTs are supported in CUDA C only for sizes that are powers of two. Therefore, we prepared CUDA C codes for allocating 16-bit and 32-bit CUDA FFT plans at the beginning of reconstruction and executing the plans on a set of tomography slices during data reconstruction by chunks. 16-bit data are additionally padded/unpadded to the power of two sizes, 32-bit data are padded to the sizes represented as 2^*a*^ × 3^*b*^ × 5^*c*^ × 7^*d*^ (*a*, *b*, *c*, *d* are positive integers) for optimal evaluation of Bluestein’s algorithm (Bluestein, 1970 ▸) in the CUDA cuFFT library.

The backprojection operator is the most computationally intensive step of the reconstruction procedure. Its direct evaluation by discretizing line integrals has a computational complexity of 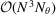 assuming that the sample size in each dimension and the total number of projection angles are of orders *N* and *N*_θ_, respectively. There exist methods for fast evaluation of the backprojection operator. The most common one is based on using the Fourier-slice theorem and using Fourier transforms on unequally spaced grids (Beylkin, 1998 ▸; Dowd *et al.*, 1999 ▸). It has complexity 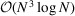 for reconstructing 3D volumes. The log-polar-based method demonstrates the same complexity (Andersson *et al.*, 2016 ▸). However, in comparison with the Fourier-based method, where interpolation-like procedures are conducted in the frequency domain, the log-polar-based method assumes interpolation in the image domain where data are substantially less oscillatory. Therefore, the log-polar-based method demonstrates accurate reconstruction results using interpolation schemes of moderate order (linear or cubic splines), whereas the Fourier-based method has to operate with exponential or other complex-type functions that can be approximated with only high-order polynomials. The log-polar-based method outperforms the Fourier-based method (Andersson *et al.*, 2016 ▸) due to the interpolation type; however, its current implementation assumes that projection data are given for equally spaced angles. In very rare cases, *e.g.* during an interlaced scanning (Mohan *et al.*, 2015 ▸), tomographic data are collected for non-equally spaced angles and the log-polar-based method is not applicable.

*TomocuPy* provides three implementations of the backprojection operator: (1) direct discretization of the backprojection line integral, (2) the Fourier-based method with exponential functions for interpolation in the frequency domain, and (3) the log-polar-based method with cubic spline interpolation in the image domain. Although the direct line discretization method is not optimal, we keep it as an option since the method can be used for computing the backprojection in a laminographic geometry (Helfen *et al.*, 2007 ▸) where the rotary stage is tilted with respect to the beam direction, yielding more efficient scanning of flat samples. Since the backprojection is the most computationally demanding part of the reconstruction, we fully implemented it with CUDA C by writing optimized codes for FFTs, CUDA raw kernels, and data handling. Users can easily switch between different backprojection methods depending on application.

### 2.3. Asynchronous data processing

Besides data processing on a GPU, tomographic reconstruction requires data transfer operations between a storage drive, CPU RAM memory and GPU memory. Non-optimal organization of data transfers among these components, especially in GPU computing, can significantly slow down the whole reconstruction pipeline, causing the GPU to be idle while waiting for new data chunk transfers to complete. Due to non-optimal organization of data transfers, significant GPU acceleration is typically visible for iterative tomography reconstructions, where data are loaded to GPU memory and tens or hundreds of iterations are performed while keeping the whole dataset in GPU memory. One-step filtered backprojection implemented on a GPU with sequential data transfers does not yield such performance gain compared with the CPU version due to relatively slow memory transfer operations. Here we organize and optimize an asynchronous processing pipeline where all data transfers are overlapped with GPU computations. In this way, the time for data transfers is effectively hidden from the total computational time required by the reconstruction step.

**Figure 1:**
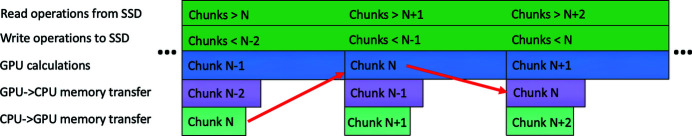
A scheme for asynchronous data processing by chunks where GPU reconstructions are overlapped with data transfers.

Figure 1 ▸ presents a scheme of the proposed asynchronous processing pipeline for data chunks. An example of execution is as follows. When data Chunk N is loaded from a storage drive, three operations are executed simultaneously: CPU–GPU memory transfer for Chunk N; GPU computations for Chunk N−1; and GPU–CPU memory transfer for Chunk N−2. After Chunk N−2 with reconstruction is copied to CPU, a write operation is executed to dump the chunk to the storage drive.

*TomocuPy* implements this optimal asynchronous pipeline in two levels. First, independent Python threads are started for (1) reading data chunks from the storage drive into a Python data queue object and (2) writing reconstructed chunks from another Python queue object to the storage drive disk. Both queue objects are stored in CPU RAM memory. The size and the number of threads for each queue are defined based on the system characteristics. To maximize the performance of parallel read/write operations we work with Intel SSD D7-P5510 Series PCIe 4.0 NVMe drives. These drives work on high-end parallel data paths for faster operations than regular SAS/SATA HDDs or SSDs, protocols of which are based on CPU cycles and are not designed to handle severe data loads.

Second, independent of read/write operations with storage drives, we overlap CPU–GPU data transfers with GPU computations by using CUDA Streams. The *CuPy* interface allows the concurrent execution of streams to be organized directly within the Python code, without writing CUDA C code. It also allows for direct allocation of pinned GPU memory, which is necessary to run data transfers and GPU computations concurrently. To implement the overlap, the pinned memory on CPU and device memory on GPU should both be allocated two data chunks and two reconstruction chunks. Three CUDA streams run simultaneously by switching between chunks; the first stream performs a data copy to the first chunk of the pinned memory, followed by transfer to the first chunk of GPU memory. The second stream performs GPU computations on the second data chunk in GPU memory (whenever it is available) and places the result in the second reconstruction chunk in GPU memory. The third stream executes a data transfer from the first reconstruction chunk in GPU memory to the first pinned memory chunk for reconstruction. The chunk is then copied to the queue for further writing to the storage drive. After processing each chunk, all streams synchronize and switch the chunk ID (0 or 1) they operate with.

**Figure 2:**
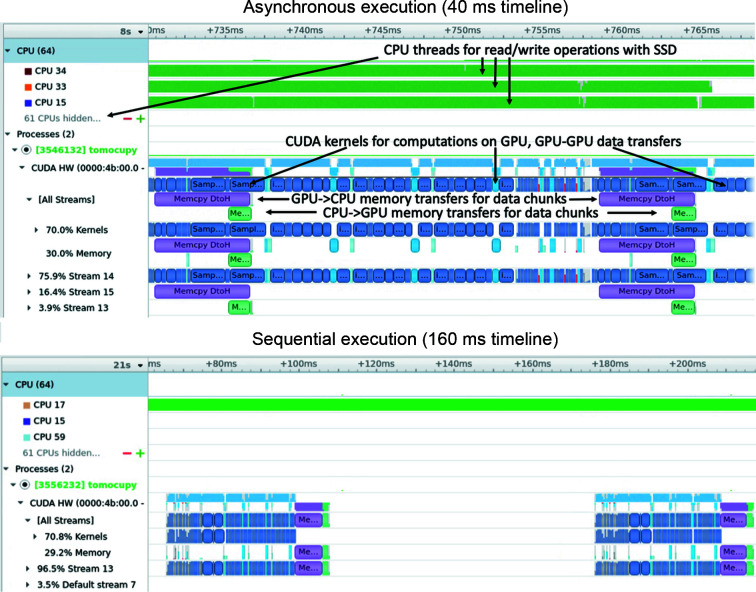
Timeline view report from the Nvidia Nsight System tool for asynchronous and sequential execution of reconstruction with *TomocuPy*. Reconstruction was performed for a 2048 × 2048 × 2048 dataset with the log-polar-based method and FP32 arithmetic.

Fig. 2 ▸ shows the timeline view report from the Nvidia Nsight System performance analysis tool that demonstrates a comparison between the asynchronous and sequential execution types. The test was performed for reconstructing a 2048 × 2048 × 2048 dataset with the log-polar-based method and FP32 arithmetic. The timeline view for the asynchronous execution is shown for 40 ms. During this time, we observe continuous data read/write operations with NVMe SSD, *i.e.* continuous CUDA kernel execution and GPU–GPU memory transfers. We also observe two GPU–CPU and two GPU–CPU memory transfers for data chunks. Since all operations are overlapped in time by using Python CPU threads and CUDA Streams, the total reconstruction time in this case can be approximately estimated only by GPU computations. In turn, the timeline view for the sequential execution with one CPU thread and one CUDA Stream shows 160 ms running time without any overlap. During this period, about 60% of the time is spent on read/write operations with an NVMe SSD in one thread; the remaining 40% is used for GPU computations and memory transfers between CPU and GPU. The left-hand panels of both reports also show that the total GPU utilization consists of about 70% CUDA kernel execution and 30% memory transfers. The total time for reconstructing a 2048 × 2048 × 2048 dataset with the asynchronous execution, as measured by the Nvidia Nsight System performance analysis tool, is approximately 2.5 times lower than for the sequential execution (8 s versus 21 s).

### 2.4. Command-line interface

To simplify the execution of tomographic reconstructions with *TomocuPy*, we have developed a command-line interface wrapping Python classes with processing functions. The command can be executed in a Unix terminal and accepts a list of parameters to customize the reconstruction procedure. The executable file is installed as part of the whole *TomocuPy* package by using pip or conda install. An example of a command line for running in an Anaconda environment and reconstructing one full tomographic dataset stored as an HDF5 file is as follows:

[Chem scheme1] 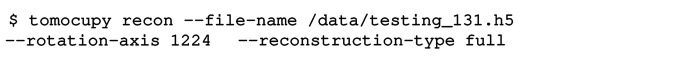


where the reconstruction parameters are submitted with the syntax 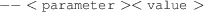 . A list of all available parameters can be obtained by running 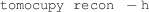 . The description of some parameters (∼20% of the full list) looks as follows:

[Chem scheme2] 
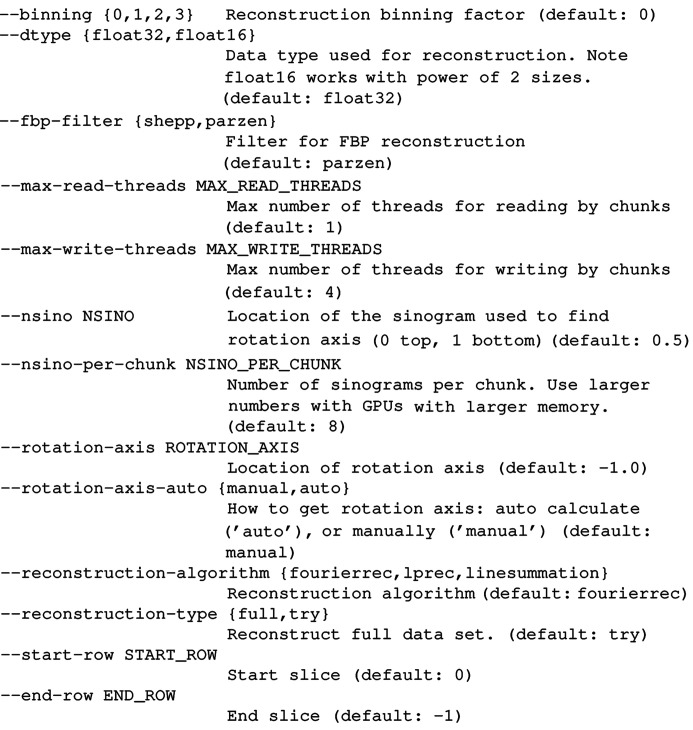


A general reconstruction procedure consists of two steps: (1) reconstruction of one slice for different rotation centers and saving reconstructed tiff files with names corresponding to these centers (parameter 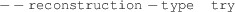 ), and (2) full reconstruction with a selected rotation center ( 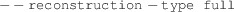 ). To select the rotation center (parameter 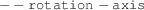 ), users open all files generated in step (1) and select the center by scrolling through different tiff files. *TomocuPy* also provides an automatic center search option ( 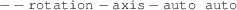 ) by using the SIFT algorithm (Lowe, 1999 ▸) to find shifts between 0 and (flipped) 180° projections.

A command-line interface for *TomocuPy* was developed to assure compatibility with the command-line interface *TomoPy-cli* (https://tomopycli.readthedocs.io) for CPU-based reconstruction. *TomoPy-cli* uses the *TomoPy* package (Gürsoy *et al.*, 2014 ▸) as a backend and implements an efficient workflow for processing tomographic data files (tiff, HDF5) from storage drives. Both packages, *TomocuPy* and *TomoPy-cli*, have the same syntax for passing parameters. They also provide the same names for most of the parameters, except method-specific parameters such as 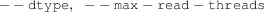 , *etc*. Likewise, the input/output format file names are identical. It is therefore not complicated to switch between two packages and compare performance and quality of reconstruction results.

It is important to note that a multi-GPU version of tomographic reconstruction is straightforward to implement because in the parallel beam geometry reconstruction is done independently for different slices through the volume. *TomocuPy* provides parameters 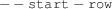 and 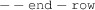 for specifying the range of slices for reconstruction, therefore multi-GPU reconstruction can be performed, for instance, by setting the environment variable 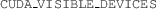 associated with the GPU number and running daemon processes in bash for each subset of slices,

[Chem scheme3] 
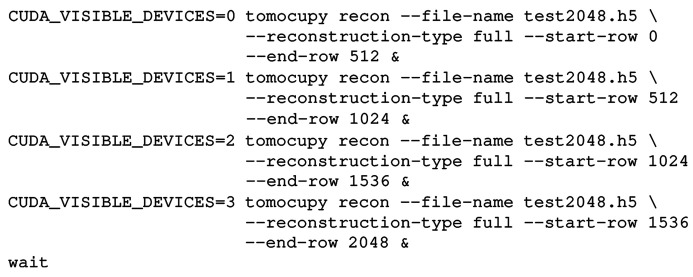
 Since the processes are independent, the total performance will be limited only by the storage and system bus speed for data transfers.

## 3. Performance and accuracy analysis

To check the reconstruction quality that the *TomocuPy* package demonstrates when processing experimental datasets, we collected tomographic projections for a sample consisting of 20–40 µm glass beads packed in a kapton tube with 4 mm diameter. The measurements were performed at the bending-magnet micro-CT beamline 2-BM (Nikitin *et al.*, 2022 ▸) of the Advanced Photon Source. The beamline was adjusted for using pink beam (polychromatic X-ray beam reflected from a grazing mirror) cutting energies higher than 30 keV, and with additional 6 mm glass filtering of low energies. Projections were acquired by a CMOS detector Oryx 5.0 MP Mono 10 GigE, 2448 × 2048 chip size, 3.45 µm pixel size, made by Teledyne FLIR LLC. The detector used a 2× magnification infinity-corrected objective by Mitutoyo resulting in 1.725 µm pixel size. The lens was focused to a 50 µm Crytur LuAG:Ce scintillator converting X-rays to visible light.

Tomographic projections were acquired in fly scanning mode, while the sample was continuously rotated for a 180° interval. In total, 2048 projections of size 2048 × 2048 (cropped field of view for the detector) were collected with 0.05 s exposure time per projection, yielding 1.7 min total acquisition time. The reconstruction procedure was performed by using three reconstruction algorithms implemented in *TomocuPy*, and by using the *Gridrec* method from *TomoPy* (with the *TomoPy-cli* interface for data pre-processing and transfers):

(1) *FourierRec* – Fourier-based method with exponential-function interpolation in the frequency domain (Beylkin, 1998 ▸), computational complexity 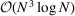 .

(2) *LpRec* – log-polar-based method with cubic interpolation in the space domain (Andersson *et al.*, 2016 ▸), computational complexity 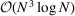 .

(3) *LineRec* – direct discretization of the line integral with linear interpolation for computing backprojection, computational complexity 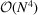 .

(4) *Gridrec* – *TomoPy* implementation of the Fourier-based method (Dowd *et al.*, 1999 ▸), computational complexity 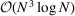 .

Here, the computational complexity is calculated assuming the number of projection angles and the object size in each dimension are of the order of *N*. All methods employed the commonly used Parzen filter for implementing filtered backprojection.

**Figure 3:**
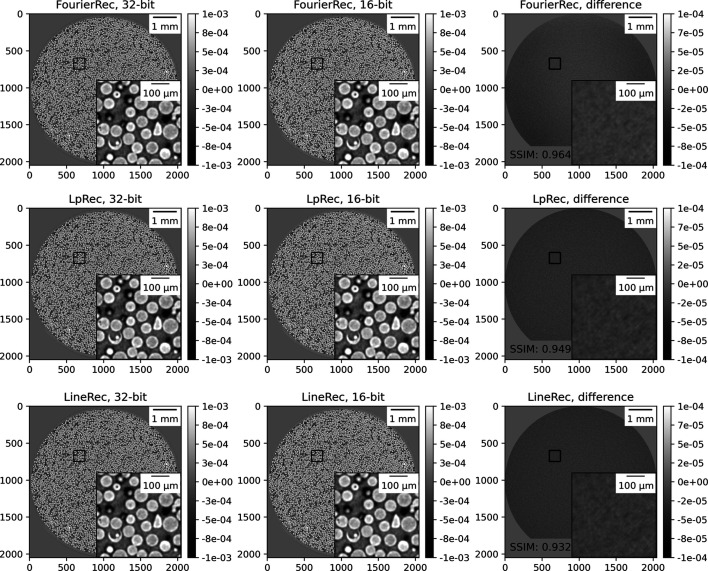
Comparison of micro glass beads reconstructions for 16- and 32-bit floating-point precision arithmetic. Inset plots show 10× zooming to the region marked by the black square. The color bar range for the difference plot is ten times smaller than for reconstructions.

Figure 3 ▸ presents a comparison for reconstructions using *TomocuPy* (*FourierRec*, *LpRec*, *LineRec*) with 32- and 16-bit floating-point arithmetic. Each image shows one reconstructed slice using different methods, together with insets showing 10× zoom-in to the region marked with the black rectangle. Visually, reconstructions for FP32 and FP16 look the same. The right-hand part of the figure shows the difference between them, *i.e.* the calculated structural similarity index (SSIM) (Wang *et al.*, 2004 ▸), quantifying the image quality degradation. SSIM is higher than 0.93 for all methods, which confirms the high quality of the FP16 results. Note that the data were collected in 12-bit precision, *i.e.* in maximum precision for most of the tomographic detectors used in fast imaging. For additional confirmation, we checked the accuracy with 16-bit synthetic Shepp–Logan phantom datasets generated as described in Section 7 of Andersson *et al.* (2016 ▸). The results confirmed that the error of the FP16 computations is negligible compared with those of FP32. We can therefore conclude that 16-bit arithmetic is sufficient for processing tomographic data, and all reconstructed volumes can be stored using twice lower amounts of memory.

As a second quality test, we compared *TomocuPy* reconstructions with those produced by the *Gridrec* method implemented in *TomoPy*. *Gridrec* is a Fourier-based method, *i.e.* uses the Fourier-slice theorem and fast evaluation of Fourier transforms on unequally spaced grids. The difference with its *TomocuPy* equivalent, called *FourierRec*, is in the interpolation kernels used for data re-gridding in the frequency domain, and oversampling factors for frequencies. *TomoPy* implementation of *Gridrec* does not include oversampling, therefore regular reconstruction contains phase wrapping artifacts. In order to minimize these artifacts, additional padding of sinograms is typically performed before the filtered backprojection operation (Marone & Stampanoni, 2012 ▸). *FourierRec* includes oversampling by a factor of two and accuracy controls in computing the backprojection integral. A detailed accuracy analysis for evaluating backprojection with the Fourier and log-polar-based methods for the Shepp–Logan phantom sample is given by Andersson *et al.* (2016 ▸). In the paper, filtered versions of the Shepp–Logan phantom, as well as corresponding projection data, are computed analytically and therefore directly used for evaluating the backprojection error for different methods. Based on the fact that the *FourierRec* method is the method with the highest accuracy in computing backprojection [based on the accuracy tests from Andersson *et al.* (2016 ▸)], we will present results for other methods in comparison with *Fourier­Rec*. Additionally, all reconstruction methods implemented in *TomocuPy* involve data padding for the filtering operation, which allows for suppressing artifacts when processing samples not fitting into the detector field of view.

**Figure 4:**
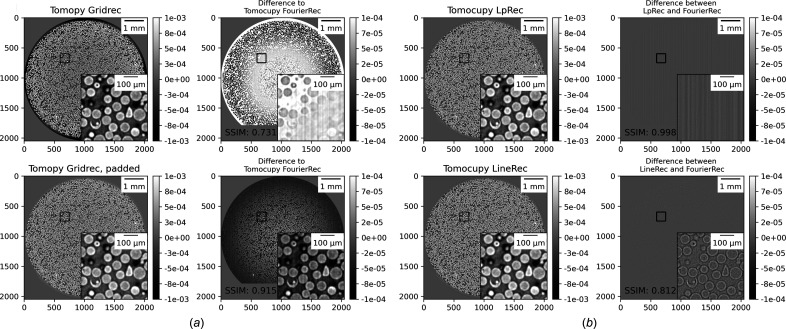
Comparison reconstruction results in 32-bit floating-point precision: (*a*) for *TomocuPy* *FourierRec* and *TomoPy Gridrec* (with and without padding of sinograms) methods, and (*b*) between *TomocuPy FourierRec*, *LpRec* and *LineRec*. Inset plots show 10× zooming to the region marked by the black square. The color bar range for the difference plots is ten times smaller than for reconstruction.

In Fig. 4 ▸(*a*) we show the difference in reconstructions between *Gridrec* from *TomoPy* and *FourierRec* from *TomocuPy*. One can see that the regular *TomoPy Gridrec* reconstruction (top row) has errors in the low-frequency components, visible as amplitude changes in the regions close to the borders. SSIM is relatively low (0.731). In turn, reconstruction with additional sinogram padding (bottom row) does not have visually observed amplitude changes; however, the difference with *TomocuPy FourierRec* still highlights errors at low frequencies. Despite the errors at low frequencies, *TomoPy* is still commonly used for reconstructing tomographic data because information given by high frequencies (small features) is more important in several applications, and it is accurately recovered with SSIM = 0.915. Note again that the accuracy of the methods implemented in *TomocuPy* was checked using analytical expressions for the Shepp–Logan phantom and its projection data (Andersson *et al.*, 2016 ▸).

In Fig. 4 ▸(*b*) we provide the difference images between *LpRec* and *FourierRec* (top) and between *LineRec* and *Fourier­Rec* (bottom). One can observe a very high accuracy of the *LpRec* method where cubic interpolations to and from log-polar coordinates are carried out in the image domain. The *LineRec* method is implemented with linear interpolation in the image domain, thus errors in high-frequency components are clearly visible. The SSIMs for these two methods are 0.998 and 0.812, respectively.

For the performance analysis, the *TomocuPy* package was tested using synthetic HDF5 datasets of different sizes. Synthetic datasets were generated for *N* 16-bit tomographic projections with *N* × *N* detector sizes, where *N* ranges from 512 to 16384. Reconstructed volumes (*N* × *N* × *N*) were obtained as sets of tiff files in 16-bit and 32-bit precision. Note that the selected projection data sizes do not satisfy the Crowther sampling criterion stating that the number of angles should be 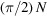 ≃ (3/2)*N* (Crowther *et al.*, 1970 ▸). In tomographic experiments this criterion is typically relaxed, and reconstruction results with acceptable quality are demonstrated for a significantly lower number of angles, *e.g.* equal to *N* or (3/4)*N*.

For completeness, we also analyzed the performance of the *TomoPy-cli* package, where all pre-processing steps and the *Gridrec* reconstruction were accelerated using multi-threaded CPU functions and the Intel Math Kernel Library. Recall that both *TomoPy-cli* and *TomocuPy* command-line interfaces have almost the same set of parameters and in most cases can be easily interchanged.

Performance tests were carried out on a machine with Intel Xeon Gold 6326 CPU @ 2.90 GHz, 1 TB DDR4 3200 memory, one Nvidia Tesla A100 with 40 GB memory, and Intel SSD D7-P5510 Series PCIe 4.0 NVMe disks of total capacity 84 TB. Installed software included Python 3.9, CuPy 10.4.0, Nvidia CUDA toolkit 11.6, and Intel Math Kernel Library Version 2022.1 (only for fast CPU-based computations in *TomoPy*).

Table 1 ▸ shows the dataset dimensions used to test the performance of *TomocuPy* methods (*FourierRec*, *LpRec*, *LineRec*) and the CPU-based *TomoPy Gridrec*.

Table 2 ▸ shows the total time to reconstruct the test datasets listed in Table 1 ▸ using *TomocuPy*’s *FourierRec* and *LpRec* methods with FP16 and FP32 precision, *TomocuPy*’s *LineRec* with FP32 precision, as well as results for the *TomoPy-cli* package where all pre-processing steps and *Gridrec* method for reconstruction are executed in FP32 precision.

There are several observations from Table 2 ▸. First, all methods allow working with very large data sizes (up to several TB on SSD), which is useful for processing data from the detectors with large sensors or from mosaic tomographic scans.

Second, we observe that FP16 computations not only reduce data sizes but also accelerate the reconstruction step by approximately 10% and 30% for *LpRec* and *FourierRec*, respectively. Double memory size reduction allowed processing data for *N* = 16384, which was not possible for 32-bit precision due to the GPU memory limit. We think that the difference between acceleration factors for the two methods (10% and 30%) is caused by the implementation of cubic B-spline interpolation procedures in the log-polar-based method [see Andersson *et al.* (2016 ▸) for details]. Hard-wired linear interpolation is implemented in GPU texture memory and works with lower than 32-bit precision due to the texture reading access organization. As a result, the performance of read/write texture access with interpolation might not be significantly different for 16- and 32-bit precision.

Third, the table shows that for large data sizes the fastest *TomocuPy* method (*LpRec*) outperforms the CPU-based *TomoPy* implementation of *Gridrec* by a factor of 33 and 29 for 16- and 32-bit precision, respectively. It has already been demonstrated that GPU is more efficient than CPU for tomographic data reconstruction [see Table 1 of Andersson *et al.* (2016 ▸)]. However, additional time for CPU–GPU data transfers and read/write operations with storage drives concealed this efficiency. With the asynchronous execution proposed in this work, the benefits of using GPU became clearly visible.

Finally, it is important to note that the computational complexity of algorithms is crucial in accelerating reconstruction algorithms. The computational complexity of all algorithms presented in Table 2 ▸ is 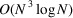 , except for *LineRec* that has complexity *O*(*N* ^4^). Although *LineRec* is also optimized and works via asynchronous execution, its reconstruction time for large data sizes is higher than that for the CPU-based *TomoPy* reconstruction. We expect to see a similar performance behavior when working with other GPU-based implementations, such as the *ASTRA Toolbox* wrapper inside *TomoPy* (Pelt *et al.*, 2016 ▸) or the *UFO* package (Vogelgesang *et al.*, 2016 ▸), where the backprojection method has computational complexity of 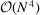 . With such complexity, the total reconstruction time for large data volumes will be mostly estimated by the GPU processing time, since the time for all data transfers increases linearly with data sizes. More accuracy and performance comparisons between the Fourier-based, log-polar, *ASTRA Toolbox* and other methods are given by Andersson *et al.* (2016 ▸).

In the previous section, we mentioned that multi-GPU reconstruction can be performed by setting the environment variable 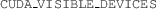 associated with the GPU number and running daemon processes in bash for subsets of slices. For demonstration, we executed reconstruction on one node of the Polaris (https://www.alcf.anl.gov/polaris) supercomputer of the Argonne Leadership Computing Facility. Compared with the workstation used for preparing Table 1 ▸, a Polaris node is equipped with a more powerful processor (AMD EPYC Milan series) and four Tesla A100 GPUs with the SXM connection interface (not PCI Express) and having high-speed HBM memory architecture. The storage is also based on NVMe PCIe v4 SSDs. From Table 3 ▸ one can see that time scaling with increasing number of GPUs is almost linear for the *FourierRec* method. In turn, the *LpRec* method demonstrates an overhead when the number of GPUs is increased from 2 to 4 (*e.g.* 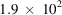 s versus 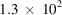 s for test dataset 5). We explain this overhead by the fact that GPU computations for *LpRec* are faster than for *FourierRec* and thus time for data management becomes more significant. Indeed, in this case four processes associated with GPUs compete with each other for the storage and system bus used for data transfers.

To provide additional performance comparison that could be relevant for the reader, in Table 4 ▸ we report reconstruction times on a regular workstation equipped with one NVidia Quadro RTX 4000 GPU and an NVMe SSD connected via PCI Express v3.0. This workstation is less expensive and therefore affordable for most tomographic beamline users. The table shows that such a workstation still demonstrates favorable performance results when processing tomographic data.

## 4. Dynamic tomography experiment with steering

To briefly demonstrate the efficacy of the *TomocuPy* package for processing data from a dynamic experiment with steering, we considered an *in situ* multi-resolution study of gas-hydrate formation inside porous media. The setup of the experiment has been given by Nikitin *et al.* (2020 ▸, 2021 ▸); multi-resolution scanning of gas-hydrates with an automatic lens-changing mechanism of the Optique Peter system (Optique-Peter, 2022 ▸) is described by Nikitin *et al.* (2022 ▸).

The whole sample was represented as a cylinder with height 2 cm and diameter 0.5 cm. For low-resolution scanning of the middle part of the sample, 1200 projections were acquired with 0.04 s exposure time per projection, which together with dark/flat-field acquisition yielded 50 s per scan. High-resolution scans with a 5× lens were acquired with 1800 angles per scan and 0.15 s exposure time. The reconstruction procedure involved dark/flat-field correction, ring removal, taking the negative logarithm, and filtered backprojection by the log-polar-based method. Additional phase-retrieval filtering (Paganin *et al.*, 2002 ▸) was applied for processing high-resolution data to enhance the gas-hydrate contrast in local tomography imaging.

For the steering demonstration, we monitored the gas-hydrate formation process in low spatial resolution (1.1× lens), detected regions with fast water flows occurring spontaneously, and automatically zoomed-in to these regions for higher-resolution (5× lens) scanning. Such automatic steering allowed us to capture the initiation and evolution of the gas-hydrate growth process inside the pore initially filled with water.

**Figure 5:**
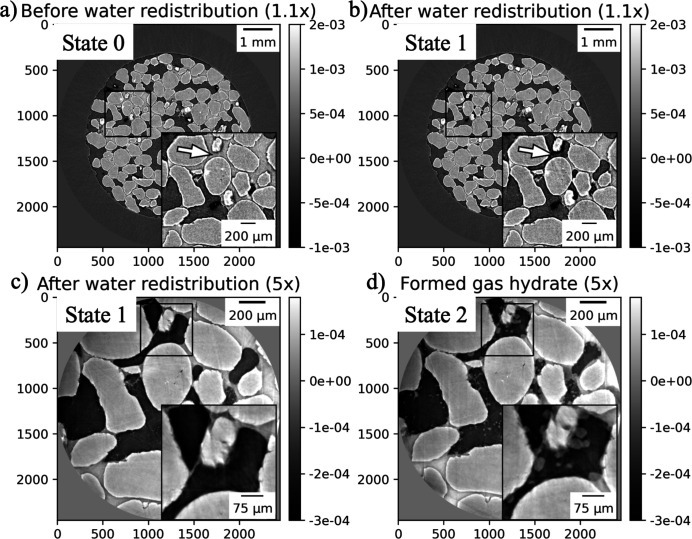
Gas-hydrate formation experiment with automatic steering (zooming to a region of interest with water outflow). (*a*, *b*) Sample states in low spatial resolution before (State 0) and after (State 1) water redistribution, respectively. (*c*) The region of interest in high spatial resolution after water redistribution (State 1). (*d*) The region of interest with formed gas hydrate (State 2). Bright color corresponds to sand grains and water solution, dark gray/black to methane gas, and light gray in high-resolution images to gas-hydrate.

The detection of regions with fast water flows was carried out after reconstructing full data volumes with *TomocuPy* and comparing them with those from the previous sample state by taking the element-wise difference. Reconstruction and region-of-interest detection took approximately 12 s, which is much less than the total scan time (50 s). Therefore the steering engine had sufficient time to select the appropriate region for high-resolution scanning with the 5× lens. Figs. 5 ▸(*a*) and 5(*b*) show slices through reconstructed volumes at low resolution for the sample state before and after water redistribution. In this figure, bright color corresponds to sand grains and water solution, and dark gray/black to methane gas. The region with water outflow is marked by white arrows. Immediately after the low-resolution scan, this region was detected and scanned at high resolution [Fig. 5 ▸(*c*)], where the hydrate structure formed on the water–gas interface can be observed in light gray color. The region was further continuously scanned until the final state (the end of experiment time) shown in Fig. 5 ▸(*d*).

In Table 5 ▸ we provide a part of the timeline for the gas-hydrate formation experiment with steering. Note that some actions, such as scanning for one state and reconstruction for another, are overlapped in time. Although this study is far from real time, it still demonstrates an example of automatic steering implementation. This dynamic study can be potentially accelerated. First, one can switch to fast data acquisition (*e.g.* with pink beam). Second, data transfers to the processing machine can be avoided by broadcasting data directly to the CPU RAM or GPU memory. Third, *TomocuPy* reconstructions can be performed with binning (Table 2 ▸ shows that reconstruction of 1024^3^ takes about 1 s). Finally, the motorized lens-changing mechanism of the Optique Peter microscope system can be replaced by a pneumatic mechanism, which will spend less than a second switching the lens as opposed to 5 s for the current motorized system.

## 5. Conclusions and outlook

By developing the *TomocuPy* package we have shown that full tomography reconstruction from a standard detector (2k × 2k sensor size), including all read/write operations with storage drives and initialization functions, can be done in less than 7 s on a single Nvidia Tesla A100 and NVMe PCIe v4 SSD. The asynchronous data processing almost completely hides the CPU–GPU data transfers time, and read/write operations with storage drives are optimized for parallel operations. Additionally, switching to 16-bit floating-point arithmetic decreased memory usage and processing times without significant reduction in reconstruction quality. The package is publicly available at https://readthedocs.org/projects/tomocupy.

Performance tests showed almost linear time scaling with increasing data sizes up to 16384^2^ slices. The linear scaling is due to efficient *TomocuPy* algorithms with low computational complexity ( 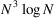 ), which becomes beneficial when working with modern detectors having large sensors. The full processing time to reconstruct a 16384^3^ volume on one GPU is approximately 1.5 h, and can be decreased with adding GPUs because tomography slices are processed independently. For comparison, a CPU-based reconstruction with an Intel Xeon Gold processor takes approximately 47 h, *i.e.* requires at least 33 computing nodes and a fast GPFS storage to demonstrate the *TomocuPy* performance. Reconstruction on one node of the Polaris supercomputer with four more powerful GPUs and fast NVMe storage took about 20 min. We note that a Tesla A100 (40 GB) has enough memory to process 16384^3^. If GPU memory is not enough, the *TomocuPy* reconstruction engine automatically switches to using unified memory (Chien *et al.*, 2019 ▸) and processes data by automatically transferring data parts to and from CPU RAM memory. However, since automatic CPU–GPU data transfers with unified memory typically show low performance, we still plan to optimize reconstruction algorithms by also processing each slice by chunks asynchronously. Specifically, we will optimize 2D FFTs and interpolation functions in *FourierRec* (interpolation to a polar grid in the frequency domain) and *LpRec* (interpolation to polar and log-polar grids in the space domain). 2D FFTs can be represented as a combination of 1D FFTs and thus computed by chunks on GPUs. Evaluation of interpolations to irregular grids can be also done by splitting all grid points into chunks that are independently processed by GPU. We expect that, by using an optimized asynchronous pipeline implemented with CUDA Streams, the overhead for CPU–GPU data transfers will be negligible, which will allow us to process huge datasets on GPUs in a reasonable time. Similar pipelines can be constructed for chunked read/write operations with storage drives if data do not fit into CPU RAM memory.

Fast 3D tomographic reconstruction with *TomocuPy* opens new possibilities for automatic steering *in situ* experiments. As a first application, we considered a geological experiment for gas-hydrate formation in porous media, where the initiation of the formation process after water redistribution was captured at high resolution inside a large sample. As the next step, we plan to conduct gas-hydrate experiments with varying cooling temperature based on the sample state. It has been shown with acoustic measurements that temperature cycles affect the hydrate growth speed (Dugarov *et al.*, 2019 ▸) and new tomography measurements may provide more details about this process. The steering mechanism could have a wide range of applications not only in geosciences but also in materials science, environmental science and medical research. We plan to study the crack formation process inside different materials. The crack will be initiated using a load cell while low-resolution projection data are continuously captured and reconstructed. The deformed regions of interest will be measured with high resolution to monitor the crack initiation in detail. Additionally, we plan to vary pressure according to the sample state obtained from reconstructions.

Although in this work we have demonstrated steering with sub-minute temporal resolution, most of the listed applications require imaging with sub-second resolution and corresponding sub-second reconstruction speeds. As Table 2 ▸ shows, such reconstruction speeds with *TomocuPy* can already be achieved for the data volumes that are smaller than 1024^3^. To steer most dynamic experiments there is no need for data reconstruction at high resolution, which means that *Tomocu­Py* can potentially be used with real-time dynamic experiments where detector data are slightly cropped or binned. However, the package needs a couple of adjustments for that. First, we need to organize streaming data processing as in Nikitin *et al.* (2022 ▸) where data are transferred directly from the detector to the processing computer over the high-speed network and where data capture to the storage drive is performed on-demand. Second, 3D reconstructed volumes will be generated by *TomocuPy* in real time and thus have to be immediately analyzed (segmented, classified, *etc*.). We envision that fast machine-learning-based techniques (probably running on an independent GPU) should optimize data analysis and generate quick automatic feedback to the acquisition system. For instance, Tekawade *et al.* (2022 ▸) have recently shown an example of real-time tomographic data analysis that can be adapted for different applications.

The *TomocuPy* package can be extended by adding new processing and reconstruction methods. New methods implemented with the Python NumPy library are directly adapted for GPU computations by switching to the Python *CuPy* library. Currently *TomocuPy* provides GPU implementations only of the one-step filtered backprojection, which is explained by the aim of having reconstructions as fast as possible. Iterative reconstruction schemes are significantly slower but they still can be added to process data more efficiently. Iterative schemes, especially those where slices are not reconstructed independently (*e.g.* 3D total variation regularization), can be implemented more efficiently with asynchronous GPU reconstruction and CPU–GPU data transfers. The same holds also for laminography reconstruction where data chunks can be organized not only in data slices but also in projections. If data volumes are too large then the asynchronous pipeline should also include read/write operations with the storage drive.

The *TomocuPy* package is in routine use at the micro-CT 2-BM and nano-CT 32-ID beamlines. Because of its easy-to-use command-line interface that has almost the same commands as the one used by *TomoPy*, the package has quickly become popular for beamline users. Data reconstruction for most experiments is currently done during the experiment beam time.

## Figures and Tables

**Table 1:** Dataset dimensions used to test the performance of *TomocuPy* methods (*FourierRec*, *LpRec*, *LineRec*) and *TomoPy Gridrec*

Test dataset	1	2	3	4	5	6
Size in each dimension, *N*	512	1024	2048	4096	8192	16384
Raw data size on SSD, 8-bit	128 MB	1 GB	8 GB	64 GB	512 GB	4 TB
Reconstruction size on SSD, 16 (32)-bit	256 (512) MB	2 (4) GB	16 (32) GB	128 (256) GB	1 (2) TB	8 (16) TB

**Table 2:** Total time to reconstruct the datasets listed in Table 1 ▸ using *TomocuPy*’s *FourierRec* and *LpRec* methods with FP16 and FP32 precision, *TomocuPy*’s *LineRec* with FP32 precision, and the *TomoPy-cli* package where all pre-processing steps and the *Gridrec* method for reconstruction are executed in FP32 precision Raw data (16-bit) and reconstructed volumes (16- or 32-bit) both have sizes *N* × *N* × *N*, assuming the number of projection angles is equal to *N*. Reconstruction time includes all parts of the processing pipeline (reading HDF5 data chunks from NVMe SSD, writing reconstructed tiff files to NVMe SSD, CPU-GPU transfers, and all CPU/GPU computations).

Test dataset	1	2	3	4	5	6
*FourierRec*, 16-bit	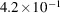 s	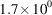 s	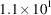 s	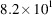 s	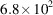 s	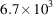 s
*FourierRec*, 32-bit	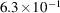 s	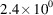 s	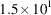 s	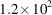 s	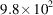 s	–
*LpRec*, 16-bit	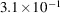 s	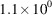 s	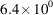 s	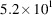 s	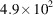 s	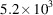 s
*LpRec*, 32-bit	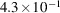 s	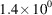 s	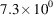 s	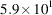 s	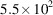 s	–
*LineRec*	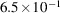 s	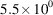 s	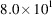 s	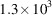 s	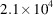 s	–
*TomoPy Gridrec*, 32-bit	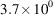 s	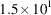 s	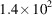 s	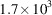 s	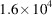 s	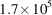 s†

† Estimated by using a lower number of chunks.

**Table 3:** Total time to reconstruct test datasets 5 (*N* = 8192) and 6 (*N* = 16384) by using one node of the Polaris supercomputer (four Tesla A100 GPUs with HBM memory, storage consisting of NVMe PCIe v4 SSDs) Reconstruction parameters are the same as in Table 2 ▸.

Test dataset	5			6		
Number of GPUs	1	2	4	1	2	4
*FourierRec*, 16-bit	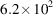 s	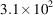 s	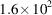 s	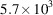 s	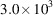 s	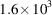 s
*LpRec*, 16-bit	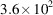 s	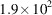 s	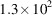 s	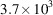 s	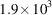 s	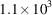 s

**Table 4:** Total time to reconstruct test datasets 1–4 (*N* = 512…4096) by using a regular workstation (one Quadro RTX 4000, 1 NVMe PCIe v3 SSD) Reconstruction parameters are the same as in Table 2 ▸.

Test dataset	1	2	3	4
*FourierRec*, 16-bit	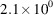 s	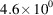 s	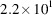 s	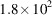 s
*LpRec*, 16-bit	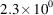 s	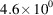 s	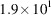 s	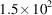 s

**Table 5:** Timeline for the gas-hydrate experiment with a steering demonstration

Time	Action
00:00–00:50	Low-resolution scan for State 0
00:50–00:53	State 0 data transfer to the processing machine
00:50–01:40	Low-resolution scan for State 1
00:53–01:01	Reconstruction for State 0
01:40–01:43	State 1 data transfer to the processing machine
01:40–01:55	Next low-resolution scan (not finished because the ROI found earlier)
	
01:43–01:51	Reconstruction for State 1
01:51–01:55	Automatic selection of the ROI by taking the difference between State 0 and State 1 reconstructions (both are in CPU memory)


01:56–02:01	Automatic lens change with the Optique Peter system and moving the sample stack motor to have the ROI in the middle of the field of view


02:02–06:34	High-resolution scan for State 1
06:34–06:38	State 1 data transfer to the processing machine
06:38–10:10	High-resolution scan for State 2
06:34–06:45	Reconstruction for State 1, including phase retrieval
…	…
